# Emergent Weyl excitations in systems of polar particles

**DOI:** 10.1038/ncomms13543

**Published:** 2016-12-12

**Authors:** Sergey V. Syzranov, Michael L. Wall, Bihui Zhu, Victor Gurarie, Ana Maria Rey

**Affiliations:** 1 Physics Department, University of Colorado, Boulder, Colorado 80309, USA; 2 JILA, NIST, University of Colorado, Boulder, Colorado 80309, USA; 3 Center for Theory of Quantum Matter, University of Colorado, Boulder, Colorado 80309, USA; 4 Joint Quantum Institute, University of Maryland, College Park, Maryland 20742, USA

## Abstract

Weyl fermions are massless chiral particles first predicted in 1929 and once thought to describe neutrinos. Although never observed as elementary particles, quasiparticles with Weyl dispersion have recently been experimentally discovered in solid-state systems causing a furore in the research community. Systems with Weyl excitations can display a plethora of fascinating phenomena and offer great potential for improved quantum technologies. Here, we show that Weyl excitations generically exist in three-dimensional systems of dipolar particles with weakly broken time-reversal symmetry (by for example a magnetic field). They emerge as a result of dipolar-interaction-induced transfer of angular momentum between the *J*=0 and *J*=1 internal particle levels. We also discuss momentum-resolved Ramsey spectroscopy methods for observing Weyl quasiparticles in cold alkaline-earth-atom systems. Our results provide a pathway for a feasible experimental realization of Weyl quasiparticles and related phenomena in clean and controllable atomic systems.

## Abstract (web summary)

Systems with Weyl excitations can display very interesting physical phenomena. Here the authors demonstrate that Weyl excitations exist generically in 3D systems of dipolar particles following angular momentum transfer, and discuss how to observe them in cold alkaline-earth-atom systems.

Recent predictions^1^,^2^ and experimental observations^3^,^4^,^5^ of Weyl semimetals in solid-state systems have instigated intensive studies of their properties, such as non-local electrodynamics and chiral anomaly^6^, topologically protected Fermi arcs on the surfaces^2^,^4^,^5^, non-Anderson disorder-driven transitions^7^,^8^,^9^,^10^,^11^,^12^,^13^ and unusual dependencies of transport properties on doping and temperature^14^,^15^,^16^. In parallel, enormous research efforts are now directed at finding Weyl excitations in new systems. A promising platform for exploring Weyl physics is tunable and fully controllable ultracold atomic gases^17^,^18^,^19^,^20^,^21^,^22^. However, henceforth proposed cold-atom realizations of Weyl quasiparticles have focussed mostly on non-interacting systems, and all have required implementations of externally imposed spin–orbit coupling through laser-assisted tunnelling schemes^17^,^18^,^19^,^20^,^21^, other optical means^23^,^24^,^25^ or external rotating fields^22^.

In this paper we demonstrate that excitations with Weyl dispersion generically emerge in three-dimensional (3D) arrays of dipolar particles in the presence of a weak magnetic field, as a result of the dipole-interactions-induced transitions between their internal angular momentum *J*=0 and *J*=1 states. These excitations exhibit the same single-particle physics as Weyl fermions^26^ but, similarly to other non-fermionic Weyl excitations^3^, their many-particle properties are expected to be different, opening alternative research directions, new functionalities and applications beyond those accessible with solid-state systems^4^,^5^.

We also show that, experimentally such excitations can be observed, for instance, in trapped alkaline-earth atoms (AEAs) in a 3D optical lattice with lattice spacings smaller than the wavelength of the electronic *J*=0 to *J*=1 transition. The simple and unique internal structure of these atoms has already lead to record levels of precision and accuracy in atomic clocks^27^. Taking advantage of the well developed spectroscopic tools to interrogate and manipulate AEAs, we propose to probe the Weyl quasiparticle dispersion and non-trivial chirality by means of momentum-resolved Ramsey spectroscopy. Our proposal opens a path for a feasible experimental realization of Weyl quasiparticles in clean and controllable atomic systems. Moreover, it lays the groundwork for the yet unexplored regime of topologically protected sensing, owing to the topological robustness of Weyl quasiparticles that could be used to push the stability and accuracy of optical lattice AEA-based clocks.

## Results

### Phenomenological argument

We assume that the system has long-lived excitations (quasiparticles) with (integer) angular momentum **J**. Due to the translational invariance, the (quasi)momentum **k** is a good quantum number. In the long-wave limit the effective quasiparticle Hamiltonian is insensitive to the details of the potential of the periodic lattice that the particles may be placed in. To preserve rotation and inversion symmetries in the absence of magnetic field the Hamiltonian has to be an even function of (**k**·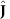) and a function of |**k**| and 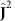. In the presence of a sufficiently weak uniform magnetic field, ***ω***, that creates a perturbation −***ω***·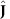 independent of **k** in the limit **k**→0, the most generic form of the quasiparticle Hamiltonian is given by


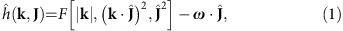


where *F* is an arbitrary function of three arguments.

The small quasimomentum **k** can be measured from any high-symmetry point in the Brillouin zone characterized by an isotropic dispersion *ξ*_**k**_=*ξ*(|**k**|) of non-interacting particles in the limit **k**→0.

For the particular case of *J*=1, the Hamiltonian (1) has nodes at momenta **K**||***ω***, such that *F*(|**K**|, |**K**|^2^, 2)±*ω*=*F*(|**K**|, 0, 2), corresponding to two intersecting branches with angular-momentum projections *J*_*z*_=0 and *J*_*z*_=1 or *J*_*z*_=−1 on the direction of magnetic field. We note that such nodes always exist for weak magnetic fields and Hamiltonians that are regular as a function of **k**.

The excitation Hamiltonian near a node is obtained by expanding the function *F* in small momentum **p**=**k**−**K**. For a 3D system, it has Weyl dispersion of the form (see ‘Methods' section):


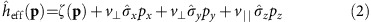


with Pauli matrices 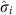 acting in the space of the respective two angular-momentum projections.

### Model

In what follows we confirm the above phenomenological argument by microscopic calculations for a 3D system of dipolar particles described by the Hamiltonian


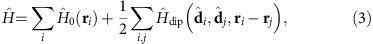


where 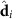 is the dipole moment operator of the *i*-th particle, and


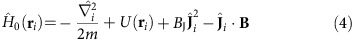


is the single-particle Hamiltonian that includes the particle kinetic energy 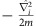 (hereinafter *ℏ*=1), the periodic potential *U*(**r**_*i*_) of the lattice that the system may be placed in, the energy *B*_J_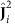 of internal levels with 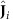 being the angular momentum of the *i*-th particle, and the interaction −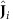
·**B** with magnetic field (measured in units of the gyromagnetic ratio) that splits the *J*=1 levels.

The most generic form of the dipole–dipole interaction, which accounts for retardation effects, is given by ref. 28 (see also ‘Methods' section)


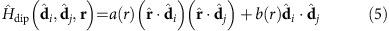


where 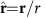; 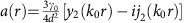 and 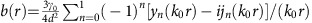 for *r*≠0, with *y*_*n*_ and *j*_*n*_ being the *n*-th-order spherical Bessel functions of the second and first kind respectively and *k*_0_—the wavevector of the *J*=0 to *J*=1 transition. The terms proportional to *y*_*n*_ describe elastic interactions between dipoles a distance *r* apart, while the terms with *j*_*n*_ account for the inelastic collective photon emission (radiation). 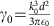 is the natural linewidth of the transition and *d* is its dipole moment. If the dipoles are much closer to one another than the wavelength of the dipole transition, *k*_0_*r*<<1, retardation effects can be ignored, and one recovers the more familiar form of the dipolar interactions, *a*(*r*)∝−3/*r*^3^, *b*(*r*)∝1/*r*^3^, common for NMR solid-state systems^29^, polar molecules^30^ and Rydberg atoms^31^,^32^.

We note that the above phenomenological derivation of the dispersion of Weyl-type quasiparticles carries over straightforwardly to other dimensions. For example, a 2D system of dipolar particles with an in-plane magnetic field hosts 2D Dirac excitations with the dispersion of monolayer graphene^33^. We emphasize that such 2D excitations are distinct from the 2D ‘chiron' excitations^34^ that exist in a perpendicular magnetic field and resemble electrons in bilayer graphene.

### Atoms in a deep lattice

**Figure 1:**
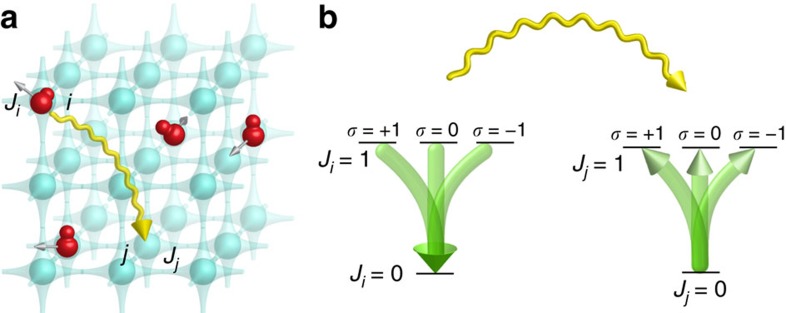
Weyl quasiparticles in 3D dipolar arrays. (**a**) Schematics of the 3D lattice potential that traps an array of dipolar particles. The lattice is deep enough to pin the particles, most of which are prepared in the *J*=0 ground state (blue spheres). Only a few particles are excited to the *J*=1 states. Dipolar interactions between the *J*=0 and *J*=1 states give raise to Weyl excitations. (**b**) Schematics of dipole mediated interactions: an excited *J*=1 state of one particle can be transferred to another particle in the *J*=0 state by dipole–dipole interactions (virtual photon exchange is shown with a yellow wiggly line). Three types of allowed processes include |00〉_*i*_|1*σ*〉_*j*_↔|1*σ*〉_*i*_|00〉_*j*_, |00〉_*i*_|10〉_*j*_↔|1, ±1〉_*i*_|00〉_*j*_ and |00〉_*i*_|1, ±1〉_*j*_↔|1, 1〉_*i*_|00〉_*j*_.

While the above phenomenological argument demonstrates the existence of Weyl quasiparticles in a generic 3D system of dipolar particles in magnetic field, below we focus on the experimentally important case of particles pinned in a deep unit-filled cubic lattice (Fig. 1a) with small lattice spacing *a*; *ak*_0_<<1.

We assume that all particles are initially prepared in the *J*_*i*_=0 state and that the energy *B*_J_ of internal levels significantly exceeds the interaction strength (usually in dipolar gases^30^,^35^ 
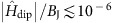
), leading to the conservation of the number of sites excited to the *J*=1 state to a good accuracy (cf. ‘Methods' section).

If an excitation with the angular momentum *J*=1 is created on site *i*, the dipole–dipole interaction can transfer it to another site *j*, possibly changing the projection of the angular momentum on the direction of the magnetic field; |1*σ*〉_*i*_→|1*σ*′〉_*j*_. The quasiparticles in the system are thus hard-core bosons corresponding to the angular-momentum degrees of freedom that hop from site to site as described by the effective Hamiltonian (see ‘Methods' section for a detailed derivation)


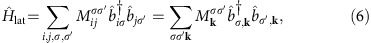



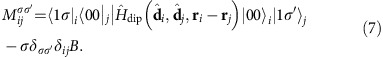


**Figure 2:**
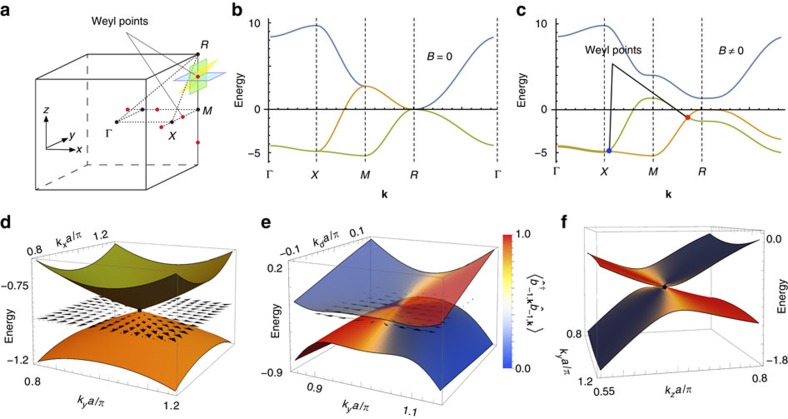
Weyl quasi-particle dispersion and eigenstates. (**a**) Brillouin zone for the simple cubic lattice. (**b**) Dispersion along high-symmetry lines in the absence of magnetic field (all energies are measured in units of (3/4)*γ*_0_/(*k*_0_*a*)^3^). (**c**) Dispersion in the presence of magnetic field *B*=*γ*_0_/(*k*_0_*a*)^3^ demonstrating the existence of Weyl nodes (red points) with linear quasiparticle dispersion near them. Each node is characterized by the monopole charge ±1. In agreement with the fermion doubling theorem^44^ (the Nielsen–Ninomiya no-go theorem), there is an even number (six) of Weyl points in the first Brillouin zone. (**d**) Dispersion in the horizontal (*k*_*x*_−*k*_*y*_) plane (shown by blue colour in panel (**a**)) containing the Weyl node near the *R* point. Quasiparticles in this plane are similar to quasiparticles in graphene and are characterized by a non-trivial Berry phase of *π*. The arrows show the pseudospin 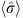 (the Pauli matrices 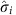 act in the space of the angular-momentum projections *J*_*z*_=0 and *J*_*z*_=−1). (**e**) Dispersion along the (yellow in panel (**a**)) plane consisting of vectors **k**=(*π*+*k*_*d*_/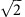, *π*+*k*_*y*_, 0.71*π*+*k*_*d*_/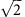) containing the Weyl point. Colour shows the weight of the |1−1〉 state in the quasiparticle eigenstate, and arrows represent the pseudospin 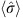. (**f**) Dispersion along the (green in panel (**a**)) vertical plane (*k*_*y*_−*k*_*z*_) containing the Weyl point near the *R* point. For each momentum **k** the colour represents the weight of the |1−1〉 state.

Due to the translational invariance, the single-excitation Hamiltonian can be diagonalised in the basis of momentum states **k**, with the results shown in Fig. 2a,c. In accordance with the above general phenomenological argument, for *B*≠0 the dispersion has Weyl nodes (six in the first Brillouin zone, Fig. 2a,c).

To demonstrate the chiral nature of Weyl quasiparticles we show in Fig. 2d–f the pseudospins 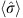 (with the Pauli matrices 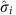 acting in the space of the *J*_*z*_=−1 and *J*_*z*_=0 angular-momentum projections) for the eigenstates with momenta **k** in the horizontal (*p*_*z*_=0), tilted (*p*_*z*_=*p*_*x*_) and vertical (*p*_*x*_=0) planes (Fig. 2a) that contain a Weyl node. Excitations in these planes are equivalent to quasiparticles in graphene, the 2D counterpart of a Weyl semimetal, and are characterized by the non-trivial Berry phase *π*. Figure 2d–f demonstrates that the pseudospins 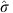 of these states are linked to their momenta **p**, measured from the Weyl node, in agreement with the effective Hamiltonian (2).

### Effects of quenched disorder and dissipation

In general, quasiparticles in interacting systems have finite lifetimes due to elastic and inelastic scattering processes. Indeed, deep optical lattices under consideration are usually not completely filled by particles and thus inherently disordered due to the randomness of the particle disptribution. Also, spontaneous and dipolar collective emission from the internal *J*=1 levels to the ground state can lead to the decay of the excitations.

**Figure 3:**
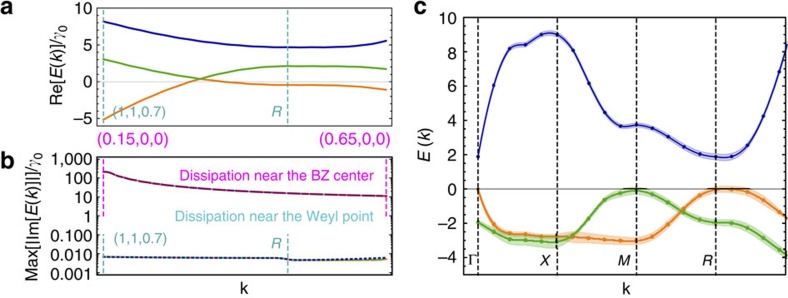
Effects of disorder and dissipation on Weyl quasiparticles. (**a**) Real part of the quasiparticle dispersion in the presence of dissipation (including spontaneous emission and collective radiative decay). The parameters of the *J*=1↔*J*=0 transition correspond to those of the electronic ^3^*P*_0_−^3^*D*_1_ levels of bosonic ^88,84^Sr atoms, trapped in a magic optical lattice potential with *a*=206.4 nm (ref. 35) at unit filling. Momentum **k** is measured in units *π*/*a*. (**b**) The upper bound on the inelastic scattering rate estimated from simulating the full quasiparticle spectra including all allowed elastic and inelastic dipolar processes. The dissipation is significantly suppressed near the Weyl node (blue line). In striking contrast, the dissipation is enhanced close to the Γ point (red line) due to the enhanced collective emission (superradiance). (**c**) Disordered case: quasiparticle dispersion for a lattice with filling fraction *f*=0.93 in the limit of small dissipation *k*_0_*a*<<1 (the energy is measured in units *γ*_0_/(*k*_0_*a*)^3^). The line thickness shows the inelastic scattering rate.

To analyse the effects of dissipation in a unit-filled lattice we compute numerically the quasiparticle dispersion for retarded dipolar interactions, equation (5), with parameters of the *J*=1 to *J*=0 transition of the electronic ^3^*P*_0_−^3^*D*_1_ levels of bosonic ^88,84^Sr atoms trapped in a magic optical lattice with *a*=206.4 nm considered in ref. 35. The wavelength and the dipole moment for this transition are 2.6 μm and *d*=4.03*D*, leading to the linewidth *γ*_0_=290 × 10^3^ s^−1^ and the dissipation parameter *ak*_0_∼0.5. Albeit quasiparticle damping in this regime is rather strong, it is significantly suppressed (by more than three orders of magnitude) near the Weyl nodes, as our simulations show, Fig. 3a,b. Our results indicate that the quasiparticle scattering in such a system would be dominated by quenched disorder rather than by collective radiative decay or spontaneous emission.

To account for the effects of disorder we evaluate numerically the quasiparticle dispersion for a lattice filling of 93%. This filling fraction could be achieved by preparing a cold bosonic Mott insulator using moderate atom numbers that allow one to suppress doubly occupied states at the trap centre. Mott insulators have already been realised with bosonic AEAs in in the ground ^1^*S*_0_ state^36^,^37^. These atoms can be excited to the desired ^3^*P*_0_ state by laser pulses^38^.

As our simulations demonstrate, the characteristic energy scales of Weyl excitations significantly exceed the elastic scattering rate, demonstrating that the excitations could be conveniently observed in current experiments.

### Experimental observation

**Figure 4:**
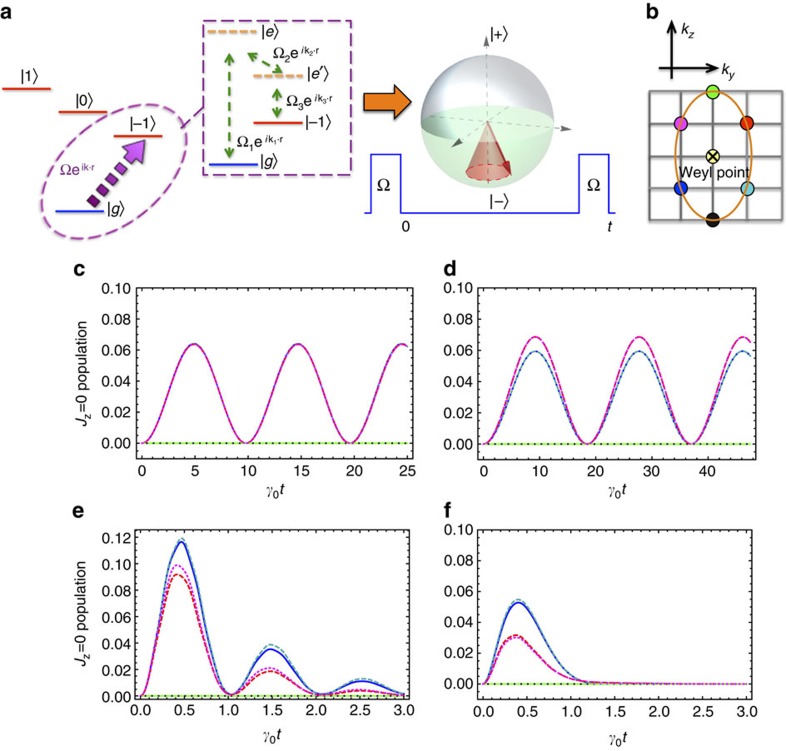
Observation of Weyl quasiparticles. (**a**) Momentum-selective Ramsey spectroscopy: interfering Raman beams create an excitation with the angular-momentum projection *J*_*z*_=−1 and with translational momentum **k** near the Weyl node (see text). After a waiting time *t* the second pulse is applied to measure the fraction of particles in the *J*_*z*_=0 angular-momentum state. (**b**) Six quasimomenta near the Weyl node. (**c**) The fraction of particles in the *J*_*z*_=0 state oscillates as a function of time *t* with the frequency (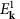
−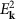)/(2*π*), where 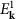
−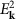 is the energy splitting between the two branches of the quasiparticle dispersion. (**d**) The oscillations in the presence of dissipation for 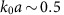. (**e**) The oscillations in a 99% randomly filled lattice. (**f**) The oscillations in a 93% filled lattice. For (**c**,**d**) a 3D cubic lattice of 100 × 100 × 100 sites was used and we took advantage of the translational symmetry. For (**e**,**f**) a 3D cubic lattice of 10 × 10 × 10 was used.

For probing the Weyl character of the excitations we propose a Ramsey protocol illustrated in Fig. 4a. After preparing a Mott insulator of particles in the *J*=0 state, a pulse of interfering Raman beams is used to create excitations in the |1,−1〉 angular-momentum state with translational momentum **k**. Here we consider the case when **k** is set to be close to the Weyl point with intersecting *J*_*z*_=0 and *J*_*z*_=−1 branches. For the proposed ^3^*P*_0_−^3^*D*_1_ electronic levels in Sr, two intermediate states |*e*〉, |*e*′〉 could be used to create the Raman pulses, imparting a net momentum to the atoms proportional to **k**=**k**_1_+**k**_2_+**k**_3_ (Fig. 4a). A possible excitation level scheme consists on using 5*s*6*s*^3^*S*_1_ and 5*s*6*p*^3^*P*_1_ as the intermediate |*e*〉 and |*e*′〉 levels respectively. After a waiting time *t*, another pulse is applied to measure the fraction of particles in the *J*_*z*_=0 angular-momentum state. Because of the interference of the two branches, this fraction oscillates with the frequency 
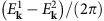
, where the energy splitting 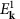
−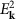 between the two branches is linear in **k** and vanishes near the Weyl node.

Another signature of the Weyl node is the strong dependency of the amplitude of such oscillations on momentum **k** near the node, as the amplitude is determined by the projection of the Bloch vector on 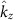 (the magnetic field direction).

In Fig. 4c–f we show the fraction of particles in the *J*_*z*_=0 state as a function of time at the end of the above described Ramsey protocol, for the six different quasi-momenta in the *k*_*z*_−*k*_*y*_ plane near the Weyl point indicated in Fig. 4b. Figure 4c shows the dynamics for an ideal unit filled lattice in the dissipationless limit *k*_0_*a*<<1. Figure 4d shows the dynamics in the presence of dissipation for the experimentally relevant scenario discussed above. The population dynamics in disordered systems is shown in Figs 4e-4f for 99% and 93% filled lattices respectively. Quasiparticles scattering on empty sites in a disordered system leads to the decay of the oscillations.

## Discussion

We demonstrated that Weyl quasiparticles generically emerge in 3D systems of polar particles in magnetic field. This opens intriguing prospects of observing chiral anomaly, non-local electrodynamics, non-Anderson disorder-driven transitions, and other fascinating phenomena in the realm of fully controllable atomic systems. We showed that observing Weyl excitations is currently possible in arrays of AEA in 3D lattices, in particular, using the ^3^*P*_0_−^3^*D*_1_ levels of bosonic Sr atoms. Other experimentally convenient schemes, that deserve further exploration, include using metastable levels of Sr or Yb atoms that can be trapped in magic lattices with spacings smaller than the wavelength^39^ or arrays of polar molecules with the rotational levels dressed to avoid the splitting of *J*=1 levels in the presence of hyperfine interactions^30^. The long lifetimes and the topological character of Weyl excitations in interacting dipolar systems also open new possibilities for implementing optical-lattice clocks with sensing capabilities beyond those of non-interacting systems.

## Methods

### Dispersion near Weyl nodes

In this work, we define the quasiparticle dispersion as the poles of the retarded Green's function averaged with respect to quenched disorder.

While long-wave quasiparticles (**k**→0) are insensitive to the details of the lattice potential, their effective Hamiltonian preserves rotation and inversion symmetries, and in the absence of magnetic field– time-reversal symmetry, with the generic form of the Hamiltonian given by equation (1) and with the vector ***ω*** parallel to the magnetic field.

We assume the existence of excitations with momentum *J*=1 and focus on the respective manifold of states in what follows. The dispersion of such excitations has three branches for each momentum **k**.

For momenta **k** parallel to ***ω*** the respective excitations have momentum projections *J*_*z*_=0 and *J*_*z*_=±1 on the direction ***ω***. The branch with *J*_*z*_=0 intersects the branch with *J*_*z*_=±1 at momenta **K**||*ω* such that


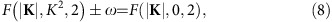


where we used that 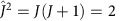 for the states under consideration.

The quasiparticle dispersion near the nodes can be found by expanding the Hamiltonian in small momenta **p**=**k**−**K**. Momentum deviation from a node along the *z* axis leads to the splitting *F*[*K*+*p*_*z*_, (*K*+*p*_*z*_)^2^, 2]±*ω*−*F*[*K*+*p*_*z*_, 0, 2] between the intersecting branches. Using that 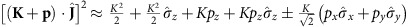, with 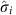 being the Pauli matrices in the space of momentum projections *J*_*z*_=+1 (*J*_*z*_=−1) and *J*_*z*_=0, we obtain the quasiparticle Hamiltonian (2) with


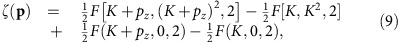



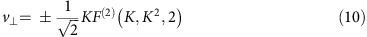



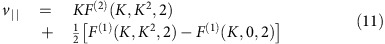


where the upper (lower) sign in equation (10) applies to the intersection of the *J*_*z*_=0 branch with *J*_*z*_=+1 (*J*_*z*_=−1), and *F*^(1)^ and *F*^(2)^ are the derivatives of the function *F* with respect to the first and the second argument.

### Generic Hamiltonian of retarded dipole–dipole interactions

The dynamics of internal transitions *J*=0↔*J*=1 in a system of *N* particles is described by the Hamiltonian


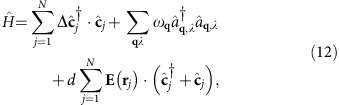


where the operator 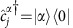 excites the *j*-th atom from the ground state |0〉 to one of the Cartesian states *α*=*x*, *y*, *z* of the *J*=1 manifold with energy Δ; *d* is the dipole moment of such a transition; 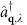 and 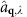 are the creation and annihilation operators of a photon with momentum **q**, frequency *ω*, and polarization *λ*; 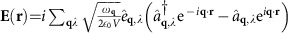 is the operator of electric field and *V* is the volume of the system.

Eliminating the electromagnetic-field modes gives in the Born–Markov approximation the master equation for for the density matrix of the particles^28^,^40^


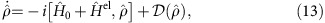


where 
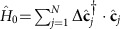
 is the Hamiltonian of the internal states of the particles, and the effective interaction Hamiltonian is given by


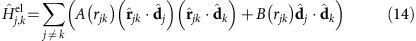


with 
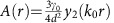
, 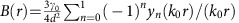 and *y*_*n*_ being the *n*-th-order spherical Bessel function of the second kind, *k*_0_ is the wavevector of the *J*=0 to *J*=1 transition, 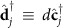, 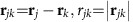, and 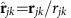.

The operator 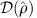 in equation (13) accounts for dissipation and is given by


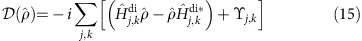


where 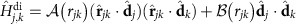, 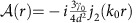, 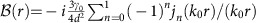, with *j*_*n*_ being the *n*-th-order spherical Bessel function of the first kind, and 
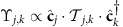
 is the so-called recycling operator^28^ that does not affect the dynamics of a single excitation and is thus omitted in the present paper. Combining the interaction 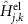 and dissipation 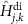 terms we obtain the effective (non-Hermitian) Hamiltonian (5) of the dipole–dipole interactions.

### Excitation dispersion in a deep lattice

As particles cannot move from site to site in a deep optical lattice, the quasiparticles are represented by the angular-momentum degrees of freedom that propagate through the system. Assuming there is one particle per site and introducing bosonic operators 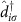 and 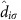 for creating and annihilating the particle state |1*σ*〉_*i*_ on site *i* with angular momentum *J*=1 and projection *σ* and the operators 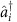 and 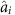 for creating and annihilating the momentum state *J*=0 on site *i*, the system Hamiltonian can be rewritten as


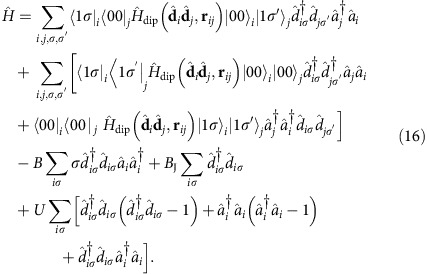


The first term in the Hamiltonian (16) is responsible for moving angular-momentum excitations from site to site; the angular-momentum state |1*σ*〉_*i*_ can be transferred by the dipole–dipole interactions from site *i* to another state |1*σ*′〉_*j*_ on site *j* that initially was in the *J*=0 state. The terms in the second sum in equation (16) change pairs of sites *i* and *j* from the *J*=0 to *J*=1 angular-momentum states or vice versa. The term ∝*B* is the Zeeman energy. The term ∝*B*_J_ accounts for the internal rotation (internal levels) of the particles. The terms ∝*U*=∞ in equation (16) enforce the hard-core constraints for the bosons created by the operators 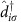 and 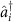, taking into account that there is one particle on each site.

In this paper we consider excitations on top of the ground state with all sites (particles) in the *J*=0 state. Exciting the internal degree of freedom of a particle on a site costs the rotation energy *B*_J_ that significantly exceeds all the other energy scales, except *U*=∞, including the matrix elements 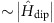 of hopping of such angular-momentum degrees of freedom between sites (for instance, for dipolar molecules and AEAs 
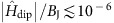
). As a result, the number of sites excited to the *J*=1 states is conserved to a good approximation, and the second sum in equation (16), that creates or annihilates pairs of *J*=1 excitations, can be neglected when considering the angular-momentum dynamics.

Therefore, the quasiparticles in the system are hard-core bosons that carry angular-momentum (*J*=1) degrees of freedom and hop from site to site as described by the effective Hamiltonian (6) and (7) with 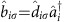.

### Details of disorder averaging

Realistic systems of particles pinned in deep optical lattices are inherently disordered due to the randomness of the spatial distribution of the particles. Each lattice site hosts either a particle with probability *f* or a vacancy with probability 1−*f*.

For a small concentration of vacancies, excitations in the system are delocalized and their dispersion is close to that in the disorder-free system but acquires a small finite imaginary part Im*E*_**k**_ due to the scattering on the vacancies.

To numerically obtain the quasiparticle spectra in such a disordered system we diagonalize the Hamiltonian 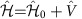, where 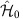 is the excitation Hamiltonian in the clean case and the operator 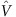 models vacancies as sites with infinite potential *V*(**r**_*i*_)=∞. We compute the retarded Green's function


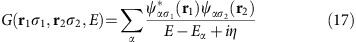


for multiple disorder realizations, where 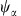 and *E*_*α*_ are the eigenfunctions and eigenenergies for a particular disorder realization, *σ*_1_ and *σ*_2_ label projections of the angular momentum *J*=1, and *η* is a small positive number introduced to ensure that the disorder-averaged Green's function 〈*G*(**r**_1_*σ*_1_, **r**_2_*σ*_2_, *E*)〉_dis_ is a smooth function of its arguments for a given number of disorder realizations. At the same time, *η* has to be chosen sufficiently small to not affect the results for the quasiparticle dispersion. The energy *E* has to be chosen close to the energies of the quasiparticles of interest.

Disorder averaging restores translational invariance, yielding an averaged Green's function that depends only on the coordinate difference **r**_2_−**r**_1_. Computing the Fourier transform of the function 〈*G*(**r**_1_*σ*_1_, **r**_2_*σ*_2_, *E*)〉_dis_ with respect to **r**_2_−**r**_1_ and diagonalising it in the angular-momentum space gives 1/(*E*−*E*_**k***n*_), where *n*=1, 2, 3 labels the dispersion branch for a given **k**, Re*E*_**k***n*_ is the quasiparticle dispersion and −2Im*E*_**k***n*_ is the scattering rate.

In this paper we perform averaging over 100 disorder realizations on a 10 × 10 × 10 cubic lattice with periodic boundary conditions for the filling fraction *f*=0.93, close to that in the recent experiments^41^,^42^,^43^. The results for the quasiparticle dispersion and scattering rates are shown in Fig. 3c.

### Data availability

The datasets generated in the current study are available from the corresponding author on reasonable request.

## Additional information

**How to cite this article:** Syzranov, S. V. *et al*. Emergent Weyl excitations in systems of polar particles. *Nat. Commun.* **7,** 13543 doi: 10.1038/ncomms13543 (2016).

**Publisher's note**: Springer Nature remains neutral with regard to jurisdictional claims in published maps and institutional affiliations.
